# Supplementary material for: The interactome of intact mitochondria by cross-linking mass spectrometry provides evidence for coexisting respiratory supercomplexes
Source: Mol Cell Proteomics. 2017 Dec 8;17(2):216–32. doi: 10.1074/mcp.RA117.000470 (PMC5795388; doi:10.1074/mcp.RA117.000470)
Supplement: Supplemental Data [file supp_RA117.000470_133922_0_supp_23978_fzffwf.zip › spectra_annotation/mito_spectra_annotation/207-2-5-1-88-1.pdf]

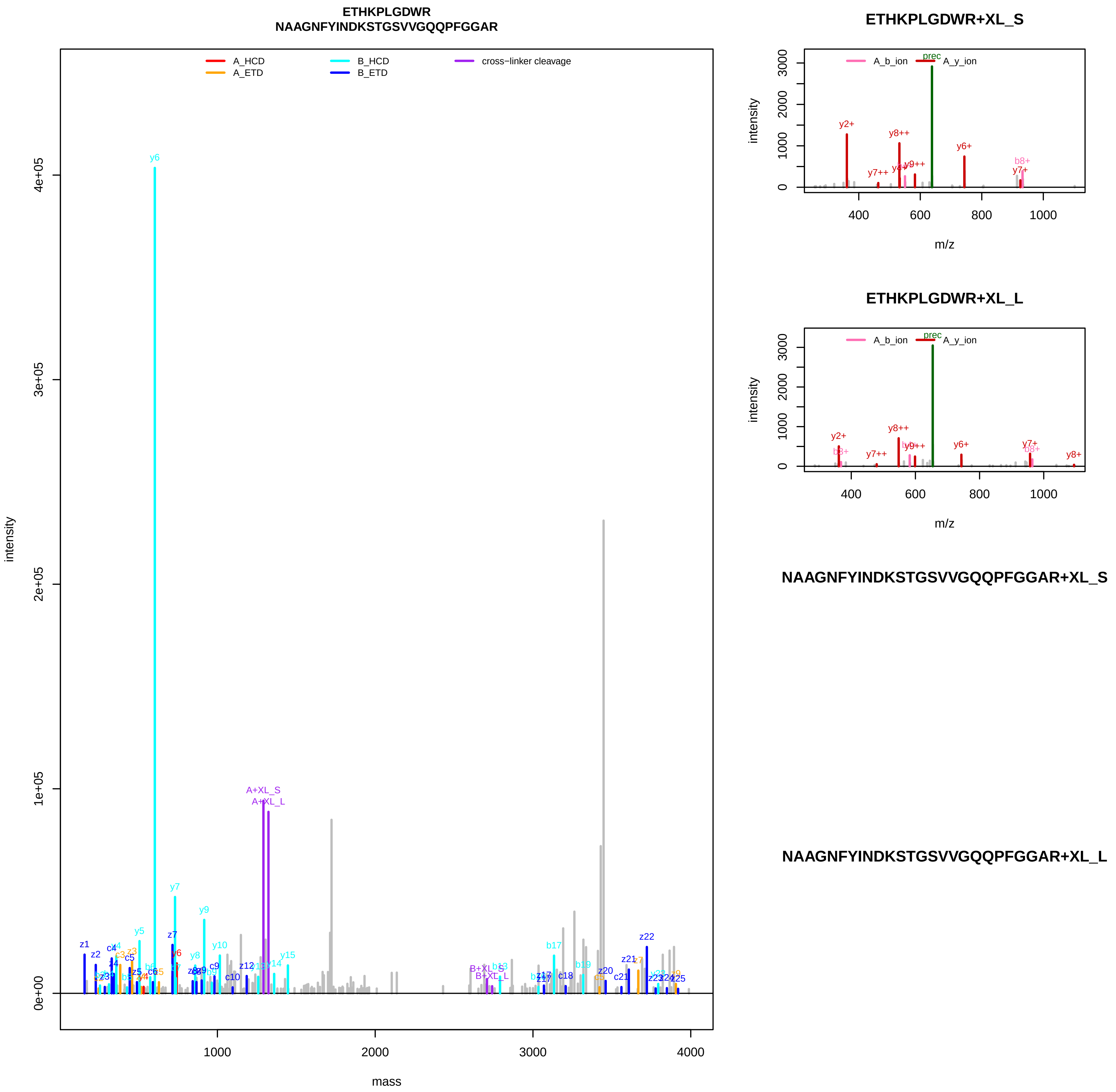

ETHKPLGDWR
NAAGNFYINDKSTGSVVGQQPFGGAR
A_HCD
A_ETD
B_HCD
B_ETD
cross−linker cleavage
intensity
mass
0e+00
1e+05
2e+05
3e+05
4e+05
1000
2000
3000
4000
y6
y7
y9
y10
y5
y15
y14
z1
z2
c4
c5
z7
z22
z21
b17
b19
c18
z20
c21
c10
z12
A+XL_S
A+XL_L
B+XL_S
B+XL_L
ETHKPLGDWR+XL_S
ETHKPLGDWR+XL_L
A_b_ion
A_y_ion
prec
m/z
y2+
y8++
y7++
y9++
y6+
y7+
b8+
y8+
NAAGNFYINDKSTGSVVGQQPFGGAR+XL_S
NAAGNFYINDKSTGSVVGQQPFGGAR+XL_L
